# Transthoracic Echocardiography-Based Prediction Model of Adverse Event Risk in Patients with COVID-19

**DOI:** 10.3390/pathophysiology29020014

**Published:** 2022-04-26

**Authors:** Elena Zelikovna Golukhova, Inessa Viktorovna Slivneva, Maxim Leonidovich Mamalyga, Damir Ildarovich Marapov, Mikhail Nikolaevich Alekhin, Mikhail Mikhailovich Rybka, Irina Vasilevna Volkovskaya

**Affiliations:** 1 A.N. Bakulev National Medical Scientific Center for Cardiovascular Surgery, Ministry of Health of the Russian Federation, 121552 Moscow, Russia; egolukhova@bakulev.ru; 2 Department of Emergency Ultrasound and Functional Diagnostics, A.N. Bakulev National Medical Scientific Center for Cardiovascular Surgery, Ministry of Health of the Russian Federation, 121552 Moscow, Russia; 3 Department of Surgical Treatment of Coronary Heart Disease, A.N. Bakulev National Medical Research Center for Cardiovascular Surgery, Ministry of Health of the Russian Federation, 121552 Moscow, Russia; mamalyga83@mail.ru; 4 Department of Public Health, Economics and Health Care Management, Kazan State Medical Academy—Branch Campus of the Federal State Budgetary Educational Institution of Further Professional Education «Russian Medical Academy of Continuous Professional Education» of the Ministry of Healthcare of the Russian Federation, 420012 Kazan, Russia; damirov@list.ru; 5 Functional Diagnostics Department of the Central Clinical Hospital with Polyclinic of the Russian Presidential Administration, 121359 Moscow, Russia; amn@mail.ru; 6 Department of Anesthesiology and Intensive Care, A.N. Bakulev National Medical Scientific Center for Cardiovascular Surgery, Ministry of Health of the Russian Federation, 121552 Moscow, Russia; mmrybka@bakulev.ru; 7 Polyclinic Department of the Institute of Coronary and Vascular Surgery, A.N. Bakulev National Medical Scientific Center for Cardiovascular Surgery, Ministry of Health of the Russian Federation, 121552 Moscow, Russia; ivvolkovskaya@bakulev.ru

**Keywords:** COVID-19, TTE, transthoracic echocardiography, predictive model, multivariable model, right ventricle free wall longitudinal strain, systolic pulmonary artery pressure

## Abstract

Cardiopulmonary disorders cause a significant increase in the risk of adverse events in patients with COVID-19. Therefore, the development of new diagnostic and treatment methods for comorbid disorders in COVID-19 patients is one of the main public health challenges. The aim of the study was to analyze patient survival and to develop a predictive model of survival in adults with COVID-19 infection based on transthoracic echocardiography (TTE) parameters. We conducted a prospective, single-center, temporary hospital-based study of 110 patients with moderate to severe COVID-19. All patients underwent TTE evaluation. The predictors of mortality we identified in univariate and multivariable models and the predictive performance of the model were assessed using receiver operating characteristic (ROC) analysis and area under the curve (AUC). The predictive model included three factors: right ventricle (RV)/left ventricle (LV) area (odds ratio (OR) = 1.048 per 1/100 increase, *p* = 0.03), systolic pulmonary artery pressure (sPAP) (OR = 1.209 per 1 mm Hg increase, *p* < 0.001), and right ventricle free wall longitudinal strain (RV FW LS) (OR = 0.873 per 1% increase, *p* = 0.036). The AUC-ROC of the obtained model was 0.925 ± 0.031 (95% confidence interval (95% CI): 0.863–0.986). The sensitivity (Se) and specificity (Sp) measures of the models at the cut-off point of 0.129 were 93.8% and 81.9%, respectively. A binary logistic regression method resulted in the development of a prognostic model of mortality in patients with moderate and severe COVID-19 based on TTE data. It may also have additional implications for early risk stratification and clinical decision making in patients with COVID-19.

## 1. Introduction

Nowadays, the rapid spread of the COVID-19 virus often results in comorbid pulmonary and cardiac dysfunction due to the high level of coronavirus tropism to respiratory system tissues and to vascular endothelium. Cardiopulmonary disorders significantly increase the risk of adverse events in COVID-19 patients. Therefore, one of the urgent public health problems is the development of new diagnostic and treatment methods for comorbid disorders in patients with COVID-19.

Coronavirus cytopathic action, inflammatory cytokine storms, and the cytokine effect on the myocardium, respiratory dysfunction and hypoxia, coagulation disorders, and disorders of the renin-angiotensin-aldosterone system are among the mechanisms underlying the development of cardiopulmonary anomalies in COVID-19 patients [1,2,3]. Increased pulmonary vascular resistance and the development of pulmonary hypertension in acute lung injury eventually lead to RV dysfunction [4]. It most often occurs as a consequence of acute respiratory distress syndrome (ARDS) [5,6] or in the context of acute pulmonary embolism following coagulation disorders and venous thromboembolism [1]. Even in the absence of cardiac abnormalities, COVID-19 progression leads to impaired central hemodynamic adaptation and exacerbates the patient’s severe conditions [7,8]. Therefore, the development of new diagnostic strategies to detect pulmonary and cardiovascular dysfunction in patients with COVID-19 is an actual task.

The aim of the study was to investigate the survival rate and to develop a predictive model of survival in adults with COVID-19 infection based on TTE data.

## 2. Materials and Methods

### 2.1. Study Design and Population Profile

We conducted a prospective, single-center, temporary hospital-based study of patients with COVID-19 infection. From 148 patients in the initial data set with laboratory-confirmed COVID-19 infection, the final sample comprised 110 patients (74%). Patients with LV systolic dysfunction (i.e., an LV ejection fraction of less than 50%, signs of myocardial asynergy, or prior myocardial infarction), valve heart disease, prior heart surgery, percutaneous revascularization, a lung injury volume of less than 25%, evidence of RV or pulmonary artery outflow tract stenosis, non-adequate transthoracic acoustic window, or hemodynamic instability at time of study were excluded from the study. The local ethics committee approved the study project during the time of the temporary hospitalization (approval code number 3, dated 25 November 2021). 

To reduce the risk of spreading infection, the study time was limited [9]. The treatment of patients differed depending on the period of the disease, clinical manifestation, leading pathogenetic syndrome, and concomitant diseases. In-hospital treatment tactics included non-pharmacological treatment (semi-bed rest, prone position of the body), oxygen therapy with possible respiratory support, and medication therapy, which included antiviral, anticoagulant, antiaggregant, anti-inflammatory, and antibacterial therapy. 

We collected and analyzed transthoracic echocardiography (TTE) data. All patients also had chest computed tomography (CT), electrocardiography, and the required set of diagnostic and laboratory tests on admission. The median age of the patients was 63.0 years [interquartile range (IQR): 51.0; 74.0], and 57.3% were male. 

The median time from the onset of the disease to hospitalization was 8 days, and the average duration of hospital stay was 13 days. Of the total patients, 11 (10.0%) were admitted to the intensive care unit (ICU) and 21 (19.1%) were transferred to the ICU due to the need for more active oxygen therapy. An analysis of the clinical profile of patients showed that most patients had a high risk of cardiovascular disease development: 81 patients (73.6%) had arterial hypertension, 20 patients (18.2%) had diabetes mellitus, and 18 patients (16.4%) had a history of cancer. Previous stroke or transient ischemic attack (present in 12.7% of the patients) and chronic obstructive pulmonary disease (COPD) (present in 10.9% of the patients) were also among the main comorbidities found. Laboratory tests showed increased levels of biomarkers of systemic inflammatory response and of thrombosis. The clinical characteristics of the patients and TTE findings are presented in Table 1.

The study sample was divided into two comparable groups depending on the outcome of the disease: survivors (*n* = 93) and non-survivors (*n* = 17).

### 2.2. Echocardiographic Analysis

Transthoracic echocardiographic examinations (TTE) were performed on a GE Vivid™ E9 ultrasound system (GE Vingmed Ultrasound AS, Horten, Norway) according to the approved protocol. Essential TTE positions (parasternal, apical, modified RV position, and subcostal) were used to visualize and evaluate the right heart. Dimensional and volumetric parameters of the left and right heart were measured in an apical four-chamber view with the calculation of the indexed parameters. LV volumes and ejection fractions were measured by biplane Simpson. Quantitative measurements were obtained according to the current recommendations of the American Society of Echocardiography and the European Association of Cardiovascular Imaging (ASE and EACVI, 2015) [10]. We recorded cine loops and images to reduce exposure time and enable subsequent remote analysis. Analysis of cine loops and images was conducted by two operators blinded to the clinical data.

All medical personnel were provided with protective equipment during the study in accordance with WHO standards [11] and the statement of protection [12]. To avoid possible virus transmission, ultrasonography was performed only on patients with confirmed COVID-19 infection. The ultrasound machine was cleaned as recommended after each patient [13,14].

LV diastolic function was assessed by measuring peak E velocity, calculation E/A, and additional parameters (E/e′, peak velocity of tricuspid regurgitation (TR), and maximum LA Vol _(i)_) [15]. Transmitral flow was assessed by pulse-wave (PW) Doppler with measuring the peak E velocity and calculation E/A ratio. Additional parameters were required in the case of an E/A ratio of ≤0.8, along with a peak E velocity of >50 cm/s, or if the E/A ratio was within the range of 0.8 to 2.0. PW tissue Doppler imaging (TDI) was used to estimate the averaged early diastolic velocity of the mitral annulus movement (e′), which was determined at the position of the septal and lateral parts of the mitral valve.

Several parameters were assessed to analyze the RV contractile function. We used an apical modified RV view for tracing the RV diastolic and systolic areas. The change of the RV fractional area (RV FAC) was calculated according to the formula:

RV FAC (%) = (RV EDA − RV ESA)/RV EDA × 100%,

where RV EDA is the end-diastolic area of RV and RV ESA is the end-systolic area of RV. The tricuspid annular plane systolic excursion (TAPSE) was determined in M-mode. The tricuspid annulus velocity (S′) was assessed by PW Doppler. To analyze the longitudinal deformation of the RV free wall—RV FW LS 2D STE (speckle-tracking echocardiography)—we used an apical modified RV view at a frame rate of >60 frames/s. The region of interest was selected with the subsequent correction of RV wall thickness. RV FW LS was expressed as an absolute value [10].

The flow of tricuspid regurgitation (TR) was assessed by color Doppler mapping, as well as by the jet density and contour characteristics in continuous-wave mode. The severity of the TR was ranged according to its significance: mild, moderate, or severe. The sPAP was determined by the peak velocity of the TR jet using Bernoulli’s equation and adding the right atrial (RA) pressure value [16]. The mean pulmonary artery pressure (meanPAP) was estimated by using the maximal pulmonary regurgitation diastolic peak velocity [17] with added RA pressure. The RA pressure was assessed by measuring the maximum diameter and degree of collapse of the inferior vena cava.

### 2.3. Reproducibility

An intraclass correlation coefficient (ICC) was used to estimate the variability within a single operator (intra-observer variability), between different operators (inter-observer variability), and at different time points (“test-retest” 2 weeks after the initial analysis). Two observers independently estimated the pre-selected images of 15 random patients.

### 2.4. Statistical Analysis

All statistical analyses were performed using SPSS Statistics v.26 software (IBM Corporation). Continuous variables were presented as median and IQR values. Categorical variables were summarized using frequencies and percentages. The Mann–Whitney U test and chi-squared test were used to compare the two groups. 

Simple logistic regression was used to assess the effect of each predictor on mortality. Next, a set of predictors based on the simple logistic regression Wald statistics was selected for further analysis in multiple logistic regression. The prognostic value of the multiple model was evaluated using ROC analysis. 

Differences were considered statistically significant for *p*-values of <0.05.

## 3. Results

Hospital mortality among patients included in the study was 15.5% (*n* = 17). Patients in the non-survivors group were older (72 years [IQR: 60; 82] vs. 62 years [IQR: 50; 73]), had higher NEWS scores (7 [IQR: 6; 8] vs. 6 [IQR: 5; 7]), and a lower SpO_2_ on admission (90% [IQR: 86; 92] vs. 93% [IQR: 92; 93]) (Table 2).

Stroke or transient ischemic attack were the most common anamnestic factors among non-survivors and were reported in 35.3% of patients vs. 8.6% in survivors (*p* = 0.008). Non-survivors had significantly higher rates of life-threatening conditions, such as developed ARDS (76.5% vs. 1.1%, *p* < 0.001), systemic inflammatory response (52.9% vs. 5.4%, *p* < 0.001), and acute heart failure (41.2% vs. 2.2%, *p* < 0.001). 

A statistically significant difference in the level of the following laboratory tests was found in non-survivors: higher level of D-dimer, *p* = 0.048; lower platelets count, *p* = 0.004; high level of C-reactive protein, *p* < 0.001; higher level of lactate dehydrogenase, *p* < 0.001; and lower lymphocytes count, *p* = 0.004.

Chest CT scans confirmed a higher volume of lung injury in non-survivor patients (80% [IQR: 64; 92] vs. 36% [IQR: 28; 48]). Invasive mechanical ventilation was used in all patients in the non-survivors group. Transfer to extracorporeal membrane oxygenation (ECMO) for severe respiratory failure only occurred in the non-survivors group. Efferent methods of detoxification were used more frequently in the group of non-survivors (17.6% vs. 2.2%, *p* = 0.026).

The median time from onset to admission did not differ between the groups. Depending on baseline severity and respiratory support, non-survivors patients were more frequently admitted to the ICU (5.4% vs. 35%), were more frequently transferred to the ICU (10.8% vs. 64%), and had increased ICU lengths of stay (*p* < 0.001).

### 3.1. Echocardiographic Analysis

TTE parameters estimation in comparative analysis in the non-survivors group presented an increase in the dimensional and volumetric parameters of the right atrium (RA), RV dilatation at basal level (42 mm vs. 39 mm, *p* = 0.004), deterioration of RV contractile function (RV FAC: 49.0% vs. 53.4%, *p* = 0.007; TAPSE: 16 mm vs. 20 mm, *p* < 0.001; S wave: 12 cm/s vs. 13 cm/s, *p* = 0.016; RV FW LS: 15.2% vs. 22.3%, *p* = 0.006), increased pulmonary pressure (sPAP: 47 mm Hg vs. 35 mm Hg, *p* < 0.001; meanPAP: 23 mm Hg vs. 15 mm Hg, *p* < 0.001), and the prevalence of moderate or severe TR (Table 3). meanPAP was not determined in 39% of patients due to imaging limitations. 

### 3.2. Logistic Regression Analysis and Prognostic Model Quality Assessment

The impact of various predictors of cardiac status according to TTE data on mortality risk in patients with COVID-19 was assessed by binary logistic regression. The results of the univariate analysis are presented in Table 4.

**Figure 1:**
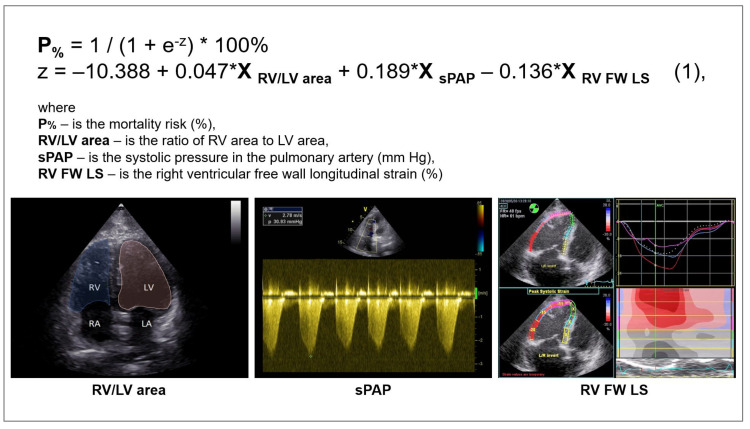
Multivariable model for predicting the risk of adverse outcomes in patients with COVID-19 infection.

**Figure 2:**
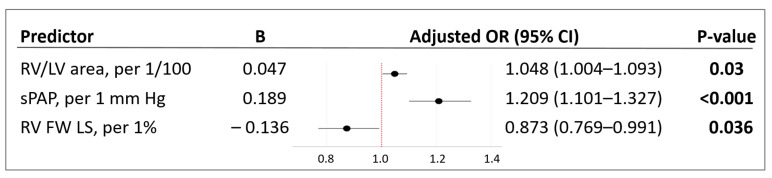
Characteristics of predictors included in the model (1). B—value of the coefficient in the equation; OR—odds ratio of mortality; 95% CI—95% confidence interval; RV/LV area—right ventricle to left ventricle area ratio; sPAP—systolic pulmonary artery pressure; RV FW LS (2D STE)—right ventricle free wall longitudinal strain (two-dimensional speckle-tracking echocardiography). Bold indicates significance (*p*-value of < 0.05).

Further, the predictors were combined in a multivariable model to predict the mortality risk in a patient with COVID-19 based on TTE parameters of the right heart. Using binary logistic regression with factor selection by exclusion, significant factors were identified, and the following model was obtained, which showed statistical significance (*p* < 0.001) (Figure 1 and Figure 2). 

**Figure 3:**
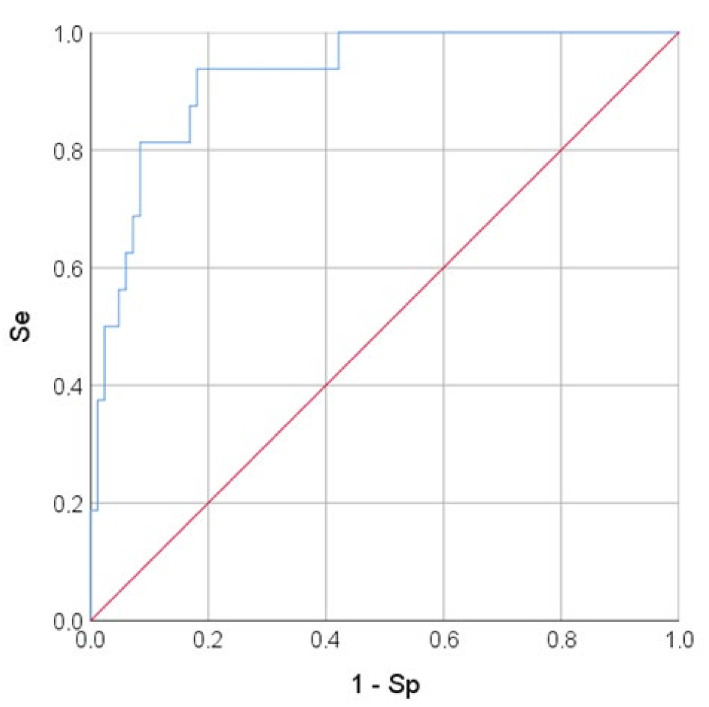
ROC-curve. The mortality risk on the values of the logistic function P%. The AUC-ROC was 0.925 ± 0.031 (95% CI: 0.863–0.986). The Se and Sp measures of model (1) at the cut-off point of 0.129 were 93.8% and 81.9%, respectively. The red line is the zero predictive value line (at Se + Sp = 100%), blue line–ROC-curve representing to the sensitivity function of the proportion of misclassified negative outcomes.

The variables RV/LV area and sPAP had a positive correlation with the mortality risk with OR = 1.048 per 1/100 increase in RV/LV area and OR = 1.209 per 1 mm Hg increase in sPAP. The longitudinal strain of the RV free wall had a negative correlation with the mortality risk (protective effect) with OR = 0.873 per 1% increase in RV FW LS. The threshold value of the logistic function *p*% was determined using the method of ROC-curve analysis. The resulting curve is shown in Figure 3.

### 3.3. Assessment of Reproducibility

The variability of the test-retest, which includes intra-server and inter-server comparisons, is presented in Table 5.

## 4. Discussion

COVID-19 is not only a respiratory disease, but also a multisystem disease, and the combination of pulmonary and extrapulmonary symptoms is typically found in patients with severe COVID-19 [18]. A number of studies [19,20] have shown that COVID-19 significantly increases the risk of mortality because it causes a complex of interrelated disorders, worsening the course of cardiovascular diseases or provoking their occurrence. 

The high level of comorbidity in the current study may reflect the age group of the patients. The median age of surviving patients was 62 years [IQR: 50; 73], and the age category of non-survivors was over 72 years [IQR: 60; 82] (*p* = 0.046). Older age has been reported previously as a risk factor for increased mortality in COVID-19 patients [21,22,23]. Patients in this age group had severe pneumonia, especially those with high blood pressure, coronary artery disease, and/or diabetes [24]. ARDS and other pulmonary complications, as well as multiple organ damage, are among the health problems reported in such patients [25].

In our study, hypertension was the most frequent anamnestic factor in both groups (71.0% in the survivors and 88.2% in the non-survivors). In addition to hypertension, diabetes mellitus, cancer, stroke or transient ischemic attack, and COPD dominated in the structure of comorbid pathology. However, statistical differences have been found only for a history of neurological disorders (stroke or transient ischemic attack). Prehospital neurological disorders were previously shown as predictors of high risk of adverse outcome [26]. The exacerbation of neurological symptoms in patients with COVID-19 may be due to the direct cytotoxic effect of the virus on the central nervous system, as well as mediated through thromboembolic and hypoxic damage, which causes cerebral edema [18].

Moreover, it has been shown in studies that cardiac rhythm disturbances occurring in COVID-19 may be due to presence of electrolyte and systemic hemodynamic disorders [27]. We found an increased incidence of atrial fibrillation in non-survivor patients with COVID-19 (*p* = 0.033). This is consistent with the results of the study conducted by Wang et al. [24], where 44% of patients with severe COVID-19 had arrhythmia.

According to Zaim et al. [19], an unfavorable COVID-19 prognosis mainly depends on the type of multiple organ failure. Analysis of the structure of multiple organ failure in our study showed that the main complications worsening the condition of patients with COVID-19 were acute respiratory distress syndrome, acute heart failure, and renal failure. These disorders exacerbated the severity of the systemic inflammatory response (increased C-reactive protein, *p* < 0.001) and hemostasis disorders (increased D-dimer, *p* = 0.048), which were also associated with an unfavorable prognosis. 

Lung injury in COVID-19 patients can contribute to the imbalance of the ventilation–perfusion ratio, which, in turn, can cause a reduction in functional residual gas volume, leading to increased pulmonary vascular resistance and the development of right heart failure [28]. According to CT findings, the volume of lung injury was significantly greater in non-survivor patients (80% [IQR: 64; 92] vs. 36% [IQR: 28; 48], *p* < 0.001).

Transthoracic echocardiography is important for the clinical assessment of patients with COVID-19, particularly those with moderate or severe disease, and it is necessary for monitoring patients with multiple areas of lung tissue consolidation in ARDS [29]. Analysis of RV size, geometry, and function is an important component of cardiac assessment and contributes to clinical decision making in patients with cardiorespiratory failure [30].

Reservoir function of the right heart compensates increasing afterload by dilatation of both the RV and the RA. Regarding TTE findings, non-survivor patients had greater RV dilatation (basal RV diameter of 42 mm [IQR: 40; 48] vs. 39 mm [IQR: 37; 43] in survivor patients). Several authors have shown that RV dilatation in patients with COVID-19 was detected more frequently than systolic dysfunction, and adverse events were more common in these patients [31,32]. The increase of pulmonary vascular resistance is accompanied by RV dilatation and an increase of both the RV area and RV to LV area ratio. Although the RV/LV area values did not differ between groups (*p* = 0.228), this parameter demonstrated prognostic significance in the univariate analysis (*p* < 0.01) and in the multivariable model (*p* < 0.03).

Pagnesi et al. [32] assessed the prognostic value of pulmonary hypertension (PH) and RV dysfunction in hospitalized patients with COVID-19 infection (*n* = 200). According to the results of this study, PH was associated with severity of COVID-19 and with worse outcomes (all-cause mortality 33.3% vs. 6.3%, *p* < 0.001). PH may serve as a better predictor of cardiopulmonary changes in COVID-19 patients than RV dysfunction [32]. However, the subsequent increase in PH will be associated with decreased RV contractility due to the limited ability to adapt to the overload.

Our study revealed decreased systolic function in non-survivors, namely RV FAC (49.0% [IQR: 42.5; 53.1] in the non-survivors group vs. 53.4% [IQR: 46.4; 60.2] in the survivors group, *p* = 0.007), TAPSE (16 mm [IQR: 16; 19] vs. 20 mm [IQR: 19; 22], *p* < 0.001), tricuspid annular S wave (12 cm/s [IQR: 9; 13] vs.13 cm/s [IQR: 12; 15], *p* = 0.016), and RV FW LS (15.2% [IQR: 11.7; 18.3] vs. 22.3% [IQR: 17.7; 26.2], *p* = 0.006). Despite the significantly lower values of RV FAC in the non-survivors group, they were higher than the reference ones [10]. Bleakley et al. [33] showed that RV FAC can be used to identify patients with RV impairment and hypothesized that radial dysfunction and not longitudinal dysfunction is a dominant phenotype; however, this study was conducted in critically ill patients (VV ECMO proportion of 42.2%).

We found the association of TAPSE with mortality with OR = 0.572, 95% CI (0.429–0.764) (*p* < 0.001). In the meta-analysis by Martha et al. [34] the prognostic value of TAPSE in patients with COVID-19 has been shown, despite the fact that the decrease of this parameter in most studies exceeded the recommended value for the diagnosis of RV dysfunction [10]. This may be due to a number of limitations, such as dependence on scan angle, loading conditions, or overestimation of TAPSE in tricuspid regurgitation. In addition, this parameter reflects RV contractility mainly at the basal level. We noted a decrease of TAPSE usually in critical patients, but even among them, TAPSE values exceeded threshold values. 

However, the use of conventional echocardiographic parameters has a limited value because of the complex shape of the RV [35,36]. It has been shown [36,37,38] that RV deformation analysis (STE) has a good predictive value as it has a higher sensitivity compared to the visual evaluation, and it can increase the predictive ability of conventional echocardiography even in presence of TR [39]. STE has been proposed for assessment of RV function due to an angle independency that leads to the increased precision in the RV dysfunction detection [10,35]. The independence of RV FW LS prognostic values from LV global systolic function in COVID-19 patients is one of the main advantages of this measure [36]. 

An important part of this study was the identification of adverse outcome predictors based on TTE data. The univariate analysis was performed by risk modelling (binary logistic regression) (Table 5). The identification of TTE risk factors for adverse outcome resulted in the construction of a multivariable prognostic model (1) (*p* < 0.001) for mortality risk prediction in patients with moderate to severe COVID-19. It included two risk factors for mortality: the sPAP and RV/LV area, and the preventive one (RV FW LS (2D STE)) (Figure 2).

Right ventricular abnormalities such as dilatation [40] and evidence of systolic dysfunction [36] have been reported as prognostic factors for adverse outcomes in patients with COVID-19. Dilation and systolic function alterations are most commonly associated with an increase in systolic pulmonary pressure [8]. We assume that the RV function changes may have a high predictive value in COVID-19, and a decrease in the ability of the RV to contract against increasing afterload leads to the development of disadaptation processes.

## 5. Limitations

The main limitation of this study is that it is a single-center study with a limited sample size. The significant heterogeneity of the data is due to large differences in populations, their ethnicity, and the lack of reference testing protocols. This work highlights the need for randomized controlled trials to confirm the results of the current study.

In addition, this study was limited to patients with moderate and severe COVID-19; patients with mild COVID-19 did not undergo TTE. 

Finally, it is not possible to identify all the causes and pathogenetic mechanisms of cardiac pathology; therefore, the ability to predict the effect of COVID-19 on the occurrence, course, and prognosis of cardiovascular pathology is limited.

## 6. Conclusions

Patients with cardiovascular disease and COVID-19 have an increased risk of mortality and adverse outcomes. The ability to predict such outcomes may be a useful tool in disease management.

In our study, we showed that TTE protocol should be focused on the assessment of right heart size, RV contractile function, and pulmonary pressure as the most sensitive parameters of RV afterload and indirect markers of lung injury severity. The developed multivariable model incorporates parameters from standard and contemporary echocardiography and can be recommended for predicting the risk of adverse outcomes in patients with COVID-19.

## Figures and Tables

**Table 1:** Clinical characteristics and TTE characteristics of the overall cohort of patients with COVID-19.

Variables		Overall (*n* = 110)
Age, years		63 [51; 74]
Male		63 (57.3%)
BSA, m/m^2^		2.01 [1.85; 2.13]
Rhythm	Sinus	89 (80.9%)
	Atrial fibrillation	18 (16.4%)
	Pacemaker (without atrial fibrillation)	3 (2.7%)
NEWS, scores		6 [5; 7]
SpO_2_ on admission to the COVID-19 hospital, %		92 [91; 93]
Comorbidities		
Hypertension		81 (73.6%)
Diabetes mellitus		20 (18.2%)
Cancer		18 (16.4%)
Encephalopathy at admission		15 (13.6%)
Stroke or transient ischemic attack		14 (12.7%)
COPD		12 (10.9%)
Bronchial asthma		9 (8.2%)
Chronic kidney disease		8 (7.3%)
Rheumatoid arthritis		3 (2.7%)
Current smoking		4 (3.6%)
TTE data		
Maximum LA Vol _(i)_, mL/m^2^		22.3 [19.1; 28.8]
LV EDI, mL/m^2^		49.6 [43.2; 58.1]
LV ESI, mL/m^2^		17.1 [14.3; 22.3]
LV SI, mL/m^2^		2.41 [2.00; 3.25]
LV EF, %		65 [60; 68]
CI, L/min/m^2^		2.44 [2.01; 3.20]
E/A		1.00 [0.80; 1.28]
E/e′		7.72 [6.15; 9.80]
Maximum RA Vol _(i)_, mL/m^2^		26.2 [19.9; 35.1]
Basal RV diameter, mm		40 [37; 43]
Mid cavitary RV diameter, mm		35 [30; 39]
RV longitudinal dimension, mm		60 [57; 66]
RV/LV area		0.64 [0.55; 0.74]
RV FAC, %		52.4 [45.1; 58.7]
TAPSE, mm		20 [18; 22]
Tricuspid annular S′ wave, cm/s (PW)		13 [11; 15]
RV FW LS, % (2D STE)		21.7 [16.2; 25.0]
sPAP, mm Hg		35 [30; 43]

Abbreviations: BSA—body surface area; NEWS—National Early Warning Score; SpO_2_—blood oxygen saturation; COPD—chronic obstructive pulmonary disease; LA—left atrium; Vol—volume; LV—left ventricle; EDI—end-diastolic volume index; ESI—end-systolic volume index; SI—stroke index; EF—ejection fraction; CI—cardiac index; RA—right atrium; RV—right ventricle; FAC—fractional area change; TAPSE—tricuspid annular plane systolic excursion; PW—pulse-wave; RV FW LS—right ventricle free wall longitudinal strain; sPAP—systolic pulmonary artery pressure. Data are expressed as number (percentage) or median [interquartile range]) values.

**Table 2:** Comparison of different parameters depending on survival status.

Variables	Survivors (*n* = 93)	Non-Survivors (*n* = 17)	*p*-Value
Age, years	62 [50; 73]	72 [60; 82]	**0.046**
Male	53 (57.0%)	10 (58.8%)	1.000
BSA, m/m^2^	1.99 [1.87; 2.10]	2.03 [1.84; 2.18]	0.738
Sinus	79 (84.9%)	10 (58.8%)	**0.019**
Atrial fibrillation	12 (12.9%)	6 (35.3%)	**0.033**
Pacemaker	2 (2.2%)	1 (5.9%)	0.399
NEWS	6 [5; 7]	7 [6; 8]	**0.047**
SpO_2_ on admission to a COVID-19 hospital, %	93 [92; 93]	90 [86; 92]	**0.002**
Comorbidities			
Hypertension	66 (71.0%)	15 (88.2%)	0.230
Diabetes mellitus	16 (17.2%)	4 (23.5%)	0.508
Cancer	16 (17.2%)	2 (11.8%)	0.734
Encephalopathy at admission	10 (10.8%)	5 (29.4%)	0.055
Stroke or transient ischemic attack	8 (8.6%)	6 (35.3%)	**0.008**
COPD	9 (9.7%)	3 (17.6%)	0.393
Bronchial asthma	6 (6.5%)	3 (17.6%)	0.143
Chronic kidney disease	6 (6.5%)	2 (11.8%)	0.607
Rheumatoid arthritis	1 (1.1%)	2 (11.8%)	0.062
Current smoking	4 (4.3%)	0	1.000
Laboratory tests			
Monocytes, ×10^9/^L	0.49 [0.34; 0.63]	0.45 [0.35; 0.63]	0.908
Neutrophils, ×10^9/^L	4.70 [3.16; 6.63]	6.07 [3.42; 8.47]	0.270
Lymphocytes, ×10^9/^L	1.15 [0.92; 1.54]	0.89 [0.60; 1.13]	**0.011**
D-dimer, ng/L	572 [342; 859]	1614 [385; 3187]	**0.048**
Hemoglobin, g/L	140.3 [131.0; 148.1]	130.9 [116.1; 142.1]	0.088
Erythrocytes, ×10^12/^L	4.83 [4.48; 5.09]	4.63 [4.36; 5.06]	0.335
White blood cells, ×10^9/^L	6.6 [4.9; 9.2]	9.5 [4.0; 11.8]	0.169
C-reactive protein, mg/L	5.5 [2.4; 9.3]	76.8 [35.3; 116.2]	**<0.001**
Platelets, ×10^9/^L	213.5 [164.2; 260.4]	148.8 [119.1; 198.1]	**0.004**
Lactate dehydrogenase, units/L	288 [227; 387]	422 [298; 665]	**<0.001**
Lung injury and oxygen therapy			
Lung tissue injury volume (CT data), %	36 [28; 48]	80 [64; 92]	**<0.001**
Nasal cannula (O_2_ up to 15 L/min)	81 (87.1%)	0	**<0.001**
High-flow nasal oxygen (AIRVO)	5 (5.4%)	0	1.000
Non-invasive ventilation	5 (5.4%)	0	1.000
Invasive mechanical ventilation	2 (2.2%)	17 (100%)	**<0.001**
VV ECMO	0	4 (23.5%)	**<0.001**
Efferent therapy methods			
Plasmapheresis	2 (2.2%)	3 (17.6%)	**0.026**
Hemosorption	2 (2.2%)	3 (17.6%)	**0.026**
Complications			
Acute respiratory distress syndrome	1 (1.1%)	13 (76.5%)	**<0.001**
Systemic inflammatory response syndrome	5 (5.4%)	9 (52.9%)	**<0.001**
Acute heart failure	2 (2.2%)	11 (41.2%)	**<0.001**
Venous thrombosis	9 (9.7%)	2 (11.8%)	0.678
Multiple organ dysfunction	1 (1.1%)	9 (52.9%)	**<0.001**
Acute kidney injury	1 (1.1%)	4 (23.5%)	**0.002**
Disseminated intravascular coagulation	0	4 (23.5%)	**<0.001**
Cerebral edema	0	3 (17.6%)	**0.003**
Gastrointestinal bleeding	0	1 (5.9%)	0.155
Haemorrhagic stroke	1 (1.1%)	0	1.000
Characteristics of admission and hospital stay			
Days from illness onset to hospital admission, days	8 [6; 11]	9 [6; 10]	0.533
ICU admission	5 (5.4%)	6 (35.3%)	**0.002**
In-hospital transfer to the ICU	10 (10.8%)	11 (64.7%)	**<0.001**
In-hospital stay, days	13 [12; 16]	12 [8; 17]	0.320
Duration of ICU stay (only ICU patients), days	1 [0; 3]	9 [7; 13]	**<0.001**

Abbreviations: BSA—body surface area; NEWS—National Early Warning Score; SpO_2_—blood oxygen saturation; COPD—chronic obstructive pulmonary disease; ICU—intensive care unit; CT—computed tomography; AIRVO—humidifier with integrated air flow generator; VV—veno-venous; ECMO—extracorporeal membrane oxygenation. Data are expressed as number (percentage) or median [interquartile range] values. Bold indicates significance (*p*-value of <0.05).

**Table 3:** Echocardiography of patients with COVID-19 infection.

Variables	Survivors (*n* = 93)	Non-Survivors (*n* = 17)	*p*-Value
Minimum LA diameter_,_ mm	39 [36; 42]	40 [38; 44]	0.388
Maximum LA diameter_,_ mm	52 [48; 56]	60 [52; 61]	**0.014**
Maximum LA Vol _(i)_, ml/m^2^	21.6 [19.1; 28.7]	25.6 [21.9; 31.0]	**0.088**
LV EDI, mL/m^2^	49.3 [43.2; 56.6]	51.4 [43.7; 68.3]	0.370
LV ESI, mL/m^2^	16.7 [14.2; 22.1]	18.0 [15.2; 28.7]	0.239
LV SI, mL/m^2^	31.8 [27.2; 36.8]	38.8 [30.2; 41.6]	0.404
LV EF, %	65.0 [60.0; 69.0]	63.0 [58.0; 65.0]	0.116
CI, l/min/m^2^	2.39 [2.00; 3.09]	3.02 [2.19; 3.71]	0.171
E/A	0.94 [0.79; 1.20]	1.40 [1.02; 1.87]	**0.009**
E/e′	7.56 [6.00; 9.14]	9.87 [8.40; 12.29]	**0.001**
LV septal wall thickness, mm	13 [12; 16]	15 [13; 17]	0.215
Characteristics of the right heart chambers			
Minimum RA diameter, mm	42 [37; 45]	44 [43; 49]	**0.007**
Maximum RA diameter, mm	51 [47; 55]	56 [51; 61]	**0.004**
Maximum RA Vol _(i)_, mL/m^2^	24.7 [19.6; 34.8]	32.2 [26.4; 42.5]	**0.002**
Basal RV diameter, mm	39 [37; 43]	42 [40; 48]	**0.004**
Mid cavitary RV diameter, mm	34 [30; 39]	36 [33; 41]	0.156
RV longitudinal dimension, mm	60 [57; 66]	64 [61; 69]	0.119
RV/LV area	0.64 [0.55; 0.73]	0.70 [0.54; 1.05]	0.228
PA trunk diameter, mm	25 [23; 27]	25 [25; 30]	0.396
RV FAC, %	53.4 [46.4; 60.2]	49.0 [42.5; 53.1]	**0.007**
TAPSE, mm	20 [19; 22]	16 [16; 19]	**<0.001**
Tricuspid annular S′ wave, cm/s (PW)	13 [12; 15]	12 [9; 13]	**0.016**
RV FW LS, % (2D STE)	22.3 [17.7; 26.2]	15.2 [11.7; 18.3]	**0.006**
RV wall thickness (subcostal), mm	5 [4; 6]	6 [5; 6]	**0.009**
Pulmonary hemodynamic parameters			
sPAP, mm Hg	35 [28; 39]	47 [42; 55]	**<0.001**
meanPAP, mm Hg	15 [11; 21]	23 [20; 31]	**<0.001**
Inferior vena cava diameter, mm	22 [19; 24]	23 [20; 24]	0.319
TR moderate to severe	14 (15.4%)	7 (43.7%)	**0.015**

Abbreviations: LA—left atrium; Vol—volume; LV—left ventricle; EDI—end-diastolic volume index; ESI—end-systolic volume index; SI—stroke index; EF—ejection fraction; CI—cardiac index; RA—right atrium; RV—right ventricle; PA—pulmonary artery; FAC—fractional area change; TAPSE—tricuspid annular plane systolic excursion; PW—pulse-wave; RV FW LS—right ventricle free wall longitudinal strain; sPAP—systolic pulmonary artery pressure; meanPAP—mean pulmonary artery pressure; TR—tricuspid regurgitation. Data are expressed as number (percentage) or median [interquartile range] values. Bold indicates significance (*p*-value of < 0.05).

**Table 4:** Results of the predictor impact assessment on mortality in patients with COVID-19 infection.

Prognostic Factor	B	OR (95% CI)	*p*-Value
Minimum diameter LA, mm	0.072	1.074 (0.969–1.191)	0.173
Maximum diameter LA, mm	0.093	1.097 (1.019–1.182)	**0.014**
Maximum LA Vol _(i)_, mL/m^2^	0.068	1.070 (1.008–1.136)	**0.026**
LV EDI, mL/m^2^	0.022	1.022 (0.983–1.064)	0.272
LV ESI, mL/m^2^	0.059	1.06 (0.980–1.147)	0.145
LV SI, mL/m^2^	0.018	1.018 (0.955–1.085)	0.585
LV EF, %	−0.060	0.942 (0.870–1.021)	0.144
CI, L/min/m^2^	0.416	1.516 (0.775–2.964)	0.224
E/A	1.099	3.001 (1.273–7.076)	**0.012**
E/e′	0.222	1.249 (1.079–1.445)	**0.003**
LV septal wall thickness, mm	0.038	1.039 (0.872–1.237)	0.670
Minimum RA diameter, mm	0.098	1.103 (1.016–1.198)	**0.019**
Maximum RA diameter, mm	0.115	1.122 (1.039–1.211)	**0.003**
Maximum RA Vol _(i)_, mL/m^2^	0.074	1.076 (1.029–1.125)	**0.001**
Basal RV diameter, mm	0.129	1.138 (1.033–1.253)	**0.009**
Mid cavitary RV diameter, mm	0.069	1.072 (0.993–1.157)	**0.075**
RV longitudinal dimension, mm	0.046	1.048 (0.978–1.122)	0.184
RV/LV area, 1/100	0.041	1.041 (1.010–1.074)	**0.010**
PA trunk diameter, mm	0.090	1.095 (0.937–1.279)	0.256
RV FAC, %	−0.069	0.933 (0.885–0.983)	**0.010**
TAPSE, mm	−0.558	0.572 (0.429–0.764)	**<0.001**
Tricuspid annular S′ wave, cm/s (PW)	−0.230	0.794 (0.656–0.962)	**0.019**
RV FW LS, % (2D STE)	−0.122	0.885 (0.808–0.970)	**0.009**
RV wall thickness (subcostal), mm	0.718	2.051 (1.123–3.745)	**0.019**
sPAP, mm Hg	0.169	1.184 (1.093–1.282)	**<0.001**
meanPAP, mm Hg	0.168	1.183 (1.082–1.293)	**<0.001**
Inferior vena cava diameter, mm	0.099	1.104 (0.906–1.346)	0.325
TR, moderate to severe/mild	1.575	4.831 (1.535–15.207)	**0.007**

Abbreviations: B—regression coefficient; OR—odds ratio; 95% CI—95% confidence interval; LA—left atrium; Vol—volume; LV—left ventricle; EDI—end-diastolic volume index; ESI—end-systolic volume index; SI—stroke index; EF—ejection fraction; CI—cardiac index; RA—right atrium; RV—right ventricle; PA—pulmonary artery; FAC—fractional area change; TAPSE—tricuspid annular plane systolic excursion; PW—pulse-wave; RV FW LS—right ventricle free wall longitudinal strain; sPAP—systolic pulmonary artery pressure; meanPAP—mean pulmonary artery pressure; TR—tricuspid regurgitation. Bold indicates significance (*p*-value of < 0.05).

**Table 5:** Results of intra-observer, inter-observer, and test-retest reproducibility analyses.

Variable	ICC (95% Confidence Interval)		
	Intra-Observer	Inter-Observer	Test-Retest
RV FW LS	0.88 (0.68–0.96)	0.84 (0.60–0.94)	0.85 (0.63–0.95)
TR velocity	0.96 (0.90–0.99)	0.96 (0.89–0.98)	0.95 (0.87–0.98)
RV area	0.97 (0.92–0.99)	0.93 (0.80–0.97)	0.95 (0.86–0.98)
LV area	0.98 (0.96–0.99)	0.97 (0.92–0.99)	0.98 (0.94–0.99)

Abbreviations: ICC—intraclass correlation coefficient; RV FW LS—right ventricular free wall longitudinal strain; TR—tricuspid regurgitation; RV—right ventricle; LV—left ventricle.

## Abbreviations

TTE	transthoracic echocardiography
AUC	area under the curve
ROC	receiver operating characteristic
RV	right ventricle
LV	left ventricle
OR	odds ratio
sPAP	systolic pulmonary artery pressure
RV FW LS	right ventricle free wall longitudinal strain
95% CI	95% confidence interval
Se	sensitivity
Sp	specificity
ARDS	acute respiratory distress syndrome
CT	computed tomography
IQR	interquartile range
ICU	intensive care unit
COPD	chronic obstructive pulmonary disease
ASE	American Society of Echocardiography
EACVI	European Association of Cardiovascular Imaging
WHO	World Health Organization
TR	tricuspid regurgitation
LA	left atrium
Vol	volume
TDI	tissue Doppler imaging
FAC	fractional area change
EDA	end-diastolic area
ESA	end-systolic area
TAPSE	tricuspid annular plane systolic excursion
2D STE	two-dimensional speckle-tracking echocardiography
PW	pulse-wave
RA	right atrium
MeanPAP	mean pulmonary artery pressure
BSA	body surface area
NEWS	patient severity rating scale
SpO_2_	blood oxygen saturation
EDI	end-diastolic volume index
ESI	end-systolic volume index
SI	stroke index
EF	ejection fraction
CI	cardiac index
ICC	intra-class correlation coefficient
AIRVO	humidifier with integrated air flow generator
VV ECMO	veno-venous extracorporeal membrane oxygenation
PA	pulmonary artery
PH	pulmonary hypertension
95% CI	95% confidence interval
